# Supplementary material for: Soluble TREM2 ameliorates tau phosphorylation and cognitive deficits through activating transgelin-2 in Alzheimer’s disease
Source: Nat Commun. 2023 Oct 21;14:6670. doi: 10.1038/s41467-023-42505-x (PMC10590452; doi:10.1038/s41467-023-42505-x)
Supplement: Supplementary file 1 — Supplementary Information [file 41467_2023_42505_MOESM1_ESM.pdf]

Supplementary Materials

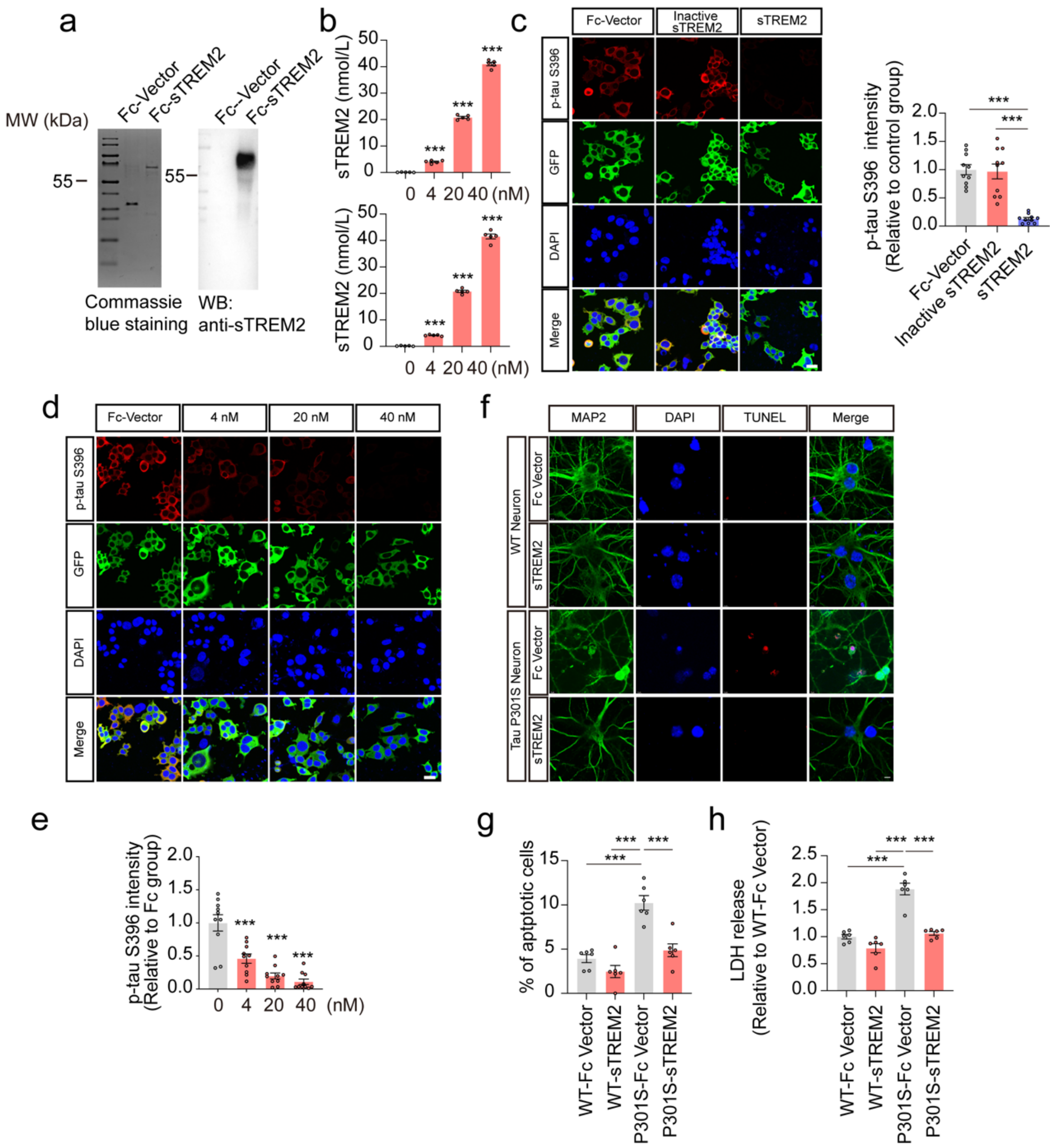

Supplementary Figure 1. sTREM2 inhibits tau phosphorylation *in vitro*. (**a**) The purity of Fc-Vector and Fc-sTREM2. Coomassie Brilliant Blue staining (left) and Western blot (right) showing purified Fc-Vector and Fc-sTREM2. (**b**) The concentrations of sTREM2 detected by ELISA in HEK293-tau cells (upper panel) or primary neurons (lower panel) (mean ± s.e.m.; n = 5 independent experiments; compared with the control group, one-way ANOVA). (**c**) p-Tau S396 immunostaining (left) and quantification (right) of HEK293-Tau cells treated with Fc, Fc-sTREM2 (40 nM), or heat-inactivated Fc-sTREM2 (40 nM) for 24 h. Scale bar, 25 µm. (mean ± s.e.m.; one-way ANOVA, n = 10 images of 5 independent experiments). (**d, e**) Western blots (d) and quantification (e) showing the phosphorylation of tau in HEK293-Tau cells treated with different concentrations of sTREM2 for 24 h. The control group (sTREM2 = 0 nM) was treated with Fc (40 nM). Scale bar, 25 µm (mean ± s.e.m.; one-way ANOVA, n = 10 images of 5 independent experiments; compared with the control

group). (**f, g**) TUNEL assay showing the anti-apoptotic effect of sTREM2 on primary neurons of tau P301S mice (mean ± s.e.m.; one-way ANOVA, n = 6 independent experiments; compared with the control group). Scale bar, 5 μm. (**h**) LDH release assay showing the protective effect of sTREM2 on the survival of tau P301S primary neurons (mean ± s.e.m.; one-way ANOVA, n = 6 independent experiments). **P* < 0.05, ***P* < 0.01, ****P* < 0.001

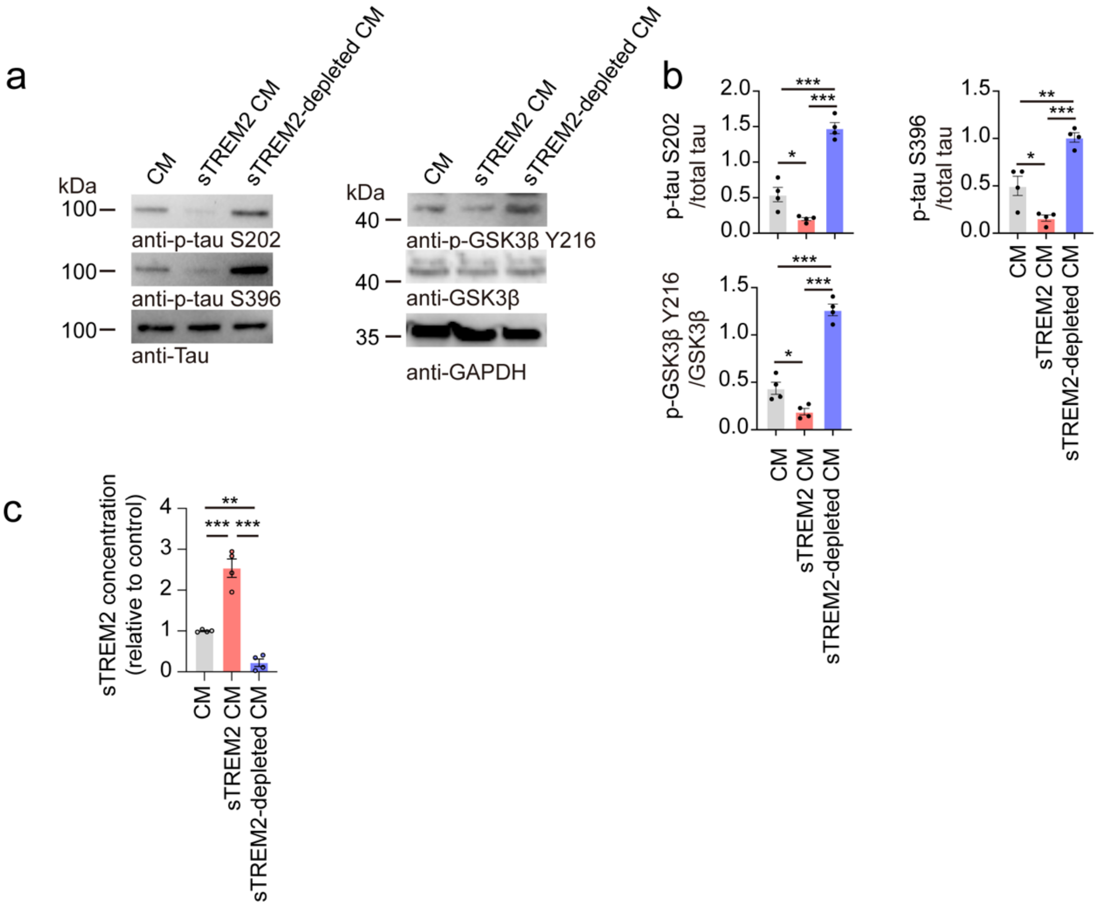

Supplementary Figure 2. Conditioned medium from BV2 cells attenuates tau phosphorylation. (**a, b**) The conditioned medium (CM) from BV2 cells transfected with AAV-sTREM2 (sTREM2 CM) or treated with anti-sTREM2 antibody (sTREM2-depleted CM) was added to HEK293-Tau cells for 24 h. Western blots (a) and quantification (b) showing the phosphorylation of tau and GSK3β. (**c**) The concentrations of sTREM2 detected by ELISA (mean ± s.e.m.; n = 4 independent experiments; **P* < 0.05, ***P* < 0.01, ****P* < 0.001, one-way ANOVA).

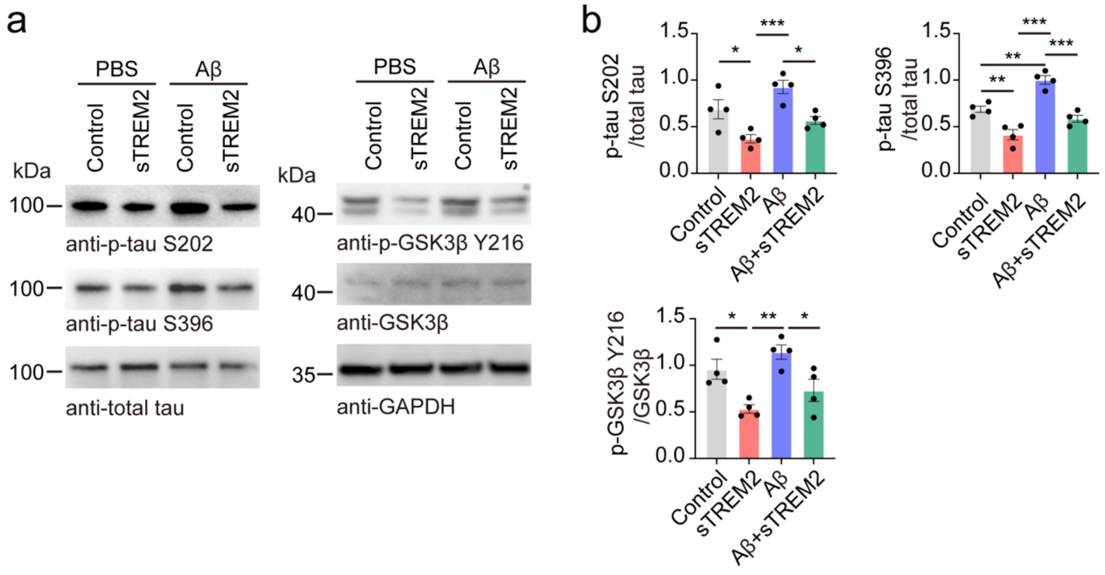

Supplementary Figure 3. sTREM2 attenuates tau phosphorylation induced by Aβ oligomers. (**a, b**) HEK293-Tau cells were treated with Aβ in the presence or absence of sTREM2 (20 nM for 24 h). Western blots (a) and quantification (b) showing the phosphorylation of tau and GSK3β in HEK293-Tau cells (mean ± s.e.m.; n = 4 independent experiments; *$P < 0.05$, **$P < 0.01$, ***$P < 0.001$, one-way ANOVA).

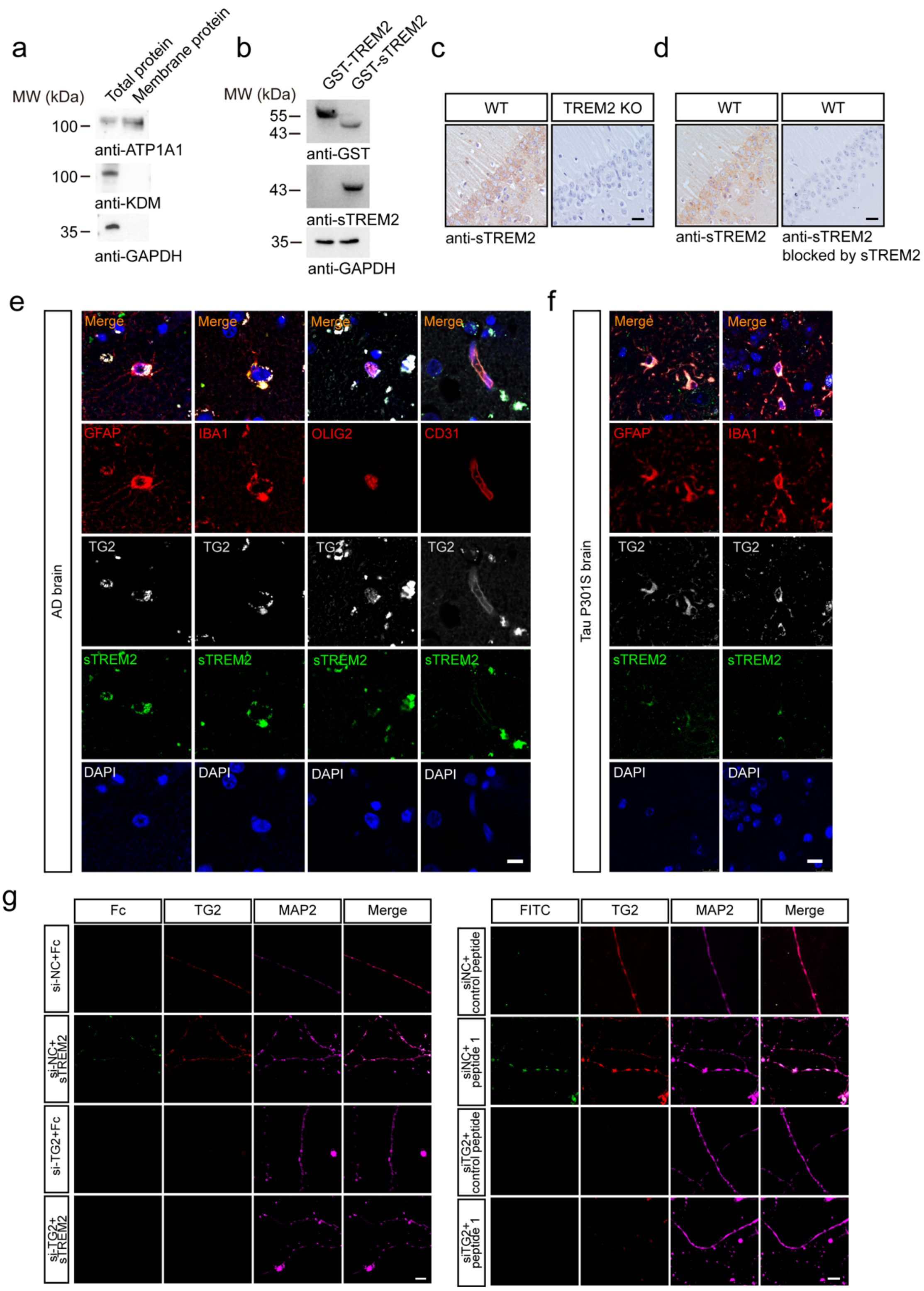

Supplementary Figure 4. sTREM2 interacts with TG2 in primary neurons. (**a**) Western blots showing the purity of extracted membrane proteins. ATP1A1, membrane marker; KDM, nuclear marker; GAPDH, cytoplasm marker. (**b**) Western blots showing the specificity of the anti-sTREM2 antibody. The antibody only recognizes sTREM2 but not full-length TREM2. (**c**) The specificity of anti-sTREM2 was verified in TREM2 knockout brain tissues. Scale bars, 20 μm. n = 4 independent experiments. (**d**) Pre-

incubation with purified sTREM2 abolished the immunosignal of the antibody. Scale bars, 20 μm. n = 4 independent experiments. (**e**) The co-localization of sTREM2 and TG2 in astrocytes (GFAP), microglia (IBA1), oligodendrocytes (OLIG2) and endothelial cells (CD31) in the hippocampus of AD brain. Scale bars, 10 μm. (**f**) The co-localization of sTREM2 and TG2 in astrocytes (GFAP) and microglia (IBA1) in the hippocampus of tau P301S mice. Scale bars, 10 μm. (**g**) TG2 knockdown abolished the binding of sTREM2 (left panels) and FITC-peptide (right panels) to neurons. Primary neurons were transfected with siNC or TG2 siRNA and incubated with Fc, Fc-sTREM2, FITC-control peptide, or FITC-peptide 1. Immunostaining showing that TG2 knockdown abolished the binding of sTREM2 to neurons. Scale bars, 5 μm.

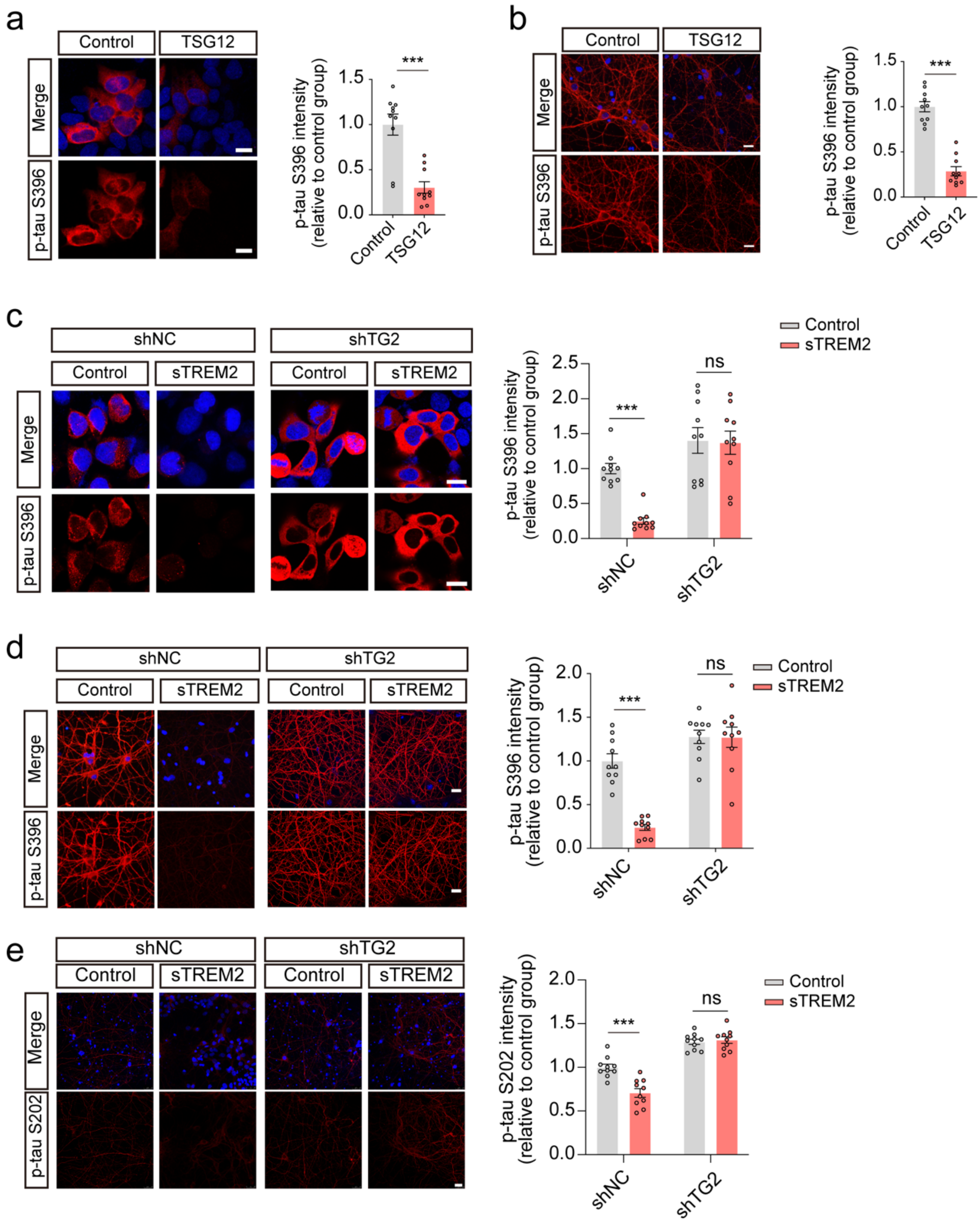

Supplementary Figure 5. sTREM2 inhibits tau phosphorylation by activating TG2. (**a**) p-Tau S396 immunostaining of HEK293-Tau cells treated with TSG12 (mean ± s.e.m.; Student's *t*-test). (**b**) p-Tau S396 immunostaining of primary cortical neurons from tau P301S mice in the presence or absence of TSG12 (mean ± s.e.m.; Student's *t*-test). (**c**) HEK293-Tau cells were pretreated with PBS or sTREM2, and then exposed to sTREM2. Shown are the p-tau S396 immunostaining (mean ± s.e.m.; two-way ANOVA). (**d**) p-tau S396 immunostaining of primary cortical neurons derived from tau P301S mice pretreated with control shRNA (sh-NC) or sh-TG2 and then exposed to

sTREM2. (mean ± s.e.m.; two-way ANOVA). (**e**) p-Tau S396 immunostaining of primary cortical neurons from WT mice pretreated with control shRNA (sh-NC) or sh-TG2, and then exposed to sTREM2. (mean ± s.e.m.; two-way ANOVA). n = 10 images of 5 independent experiments, ***P < 0.001. Scale bars are shown, 15 μm in (A) and 20 μm in (B, C, D, E).

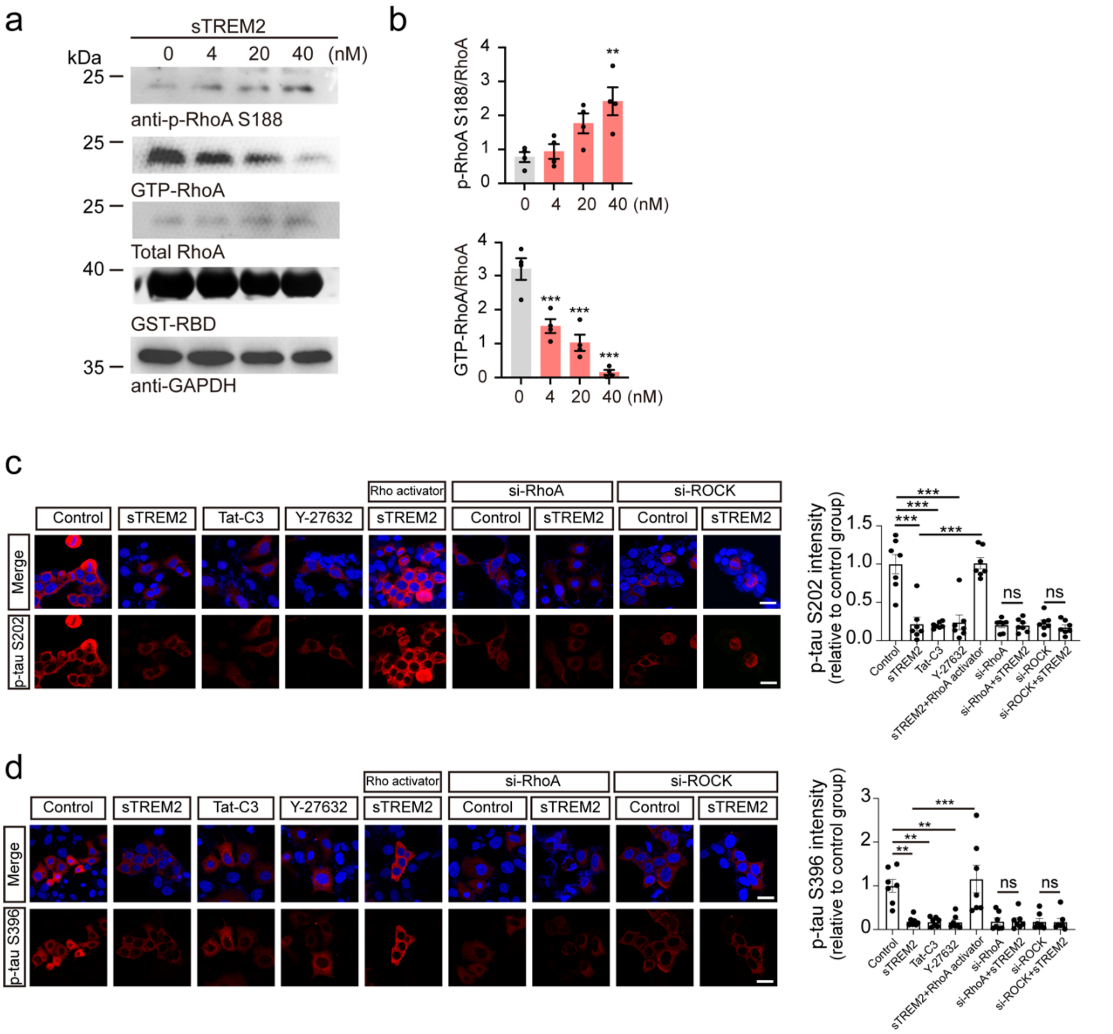

Supplementary Figure 6. The RhoA/ROCK pathway mediates the inhibitory effect of sTREM2 on tau phosphorylation. (**a, b**) Western blots and quantification showing the phosphorylation and activity of RhoA in HEK293-Tau cells treated with different concentrations of sTREM2 for 24 h (mean ± s.e.m.; one-way ANOVA, n = 4 independent experiments; compared with the control group, **P < 0.01, ***P < 0.001). (**c, d**) HEK293-tau cells were treated with sTREM2 (20 nM), RhoA inhibitor (Tat-C3, 0.1 μg/ml), ROCK inhibitor (Y-27632, 25 nM), RhoA activator (1 U/ml), or RhoA/ROCK siRNA. Shown are the p-tau S202 (c) and S396 (d) immunostaining (mean ± s.e.m.; one-way ANOVA, n = 7 independent experiments; **P < 0.01, ***P < 0.001). Scale bars, 25 μm.

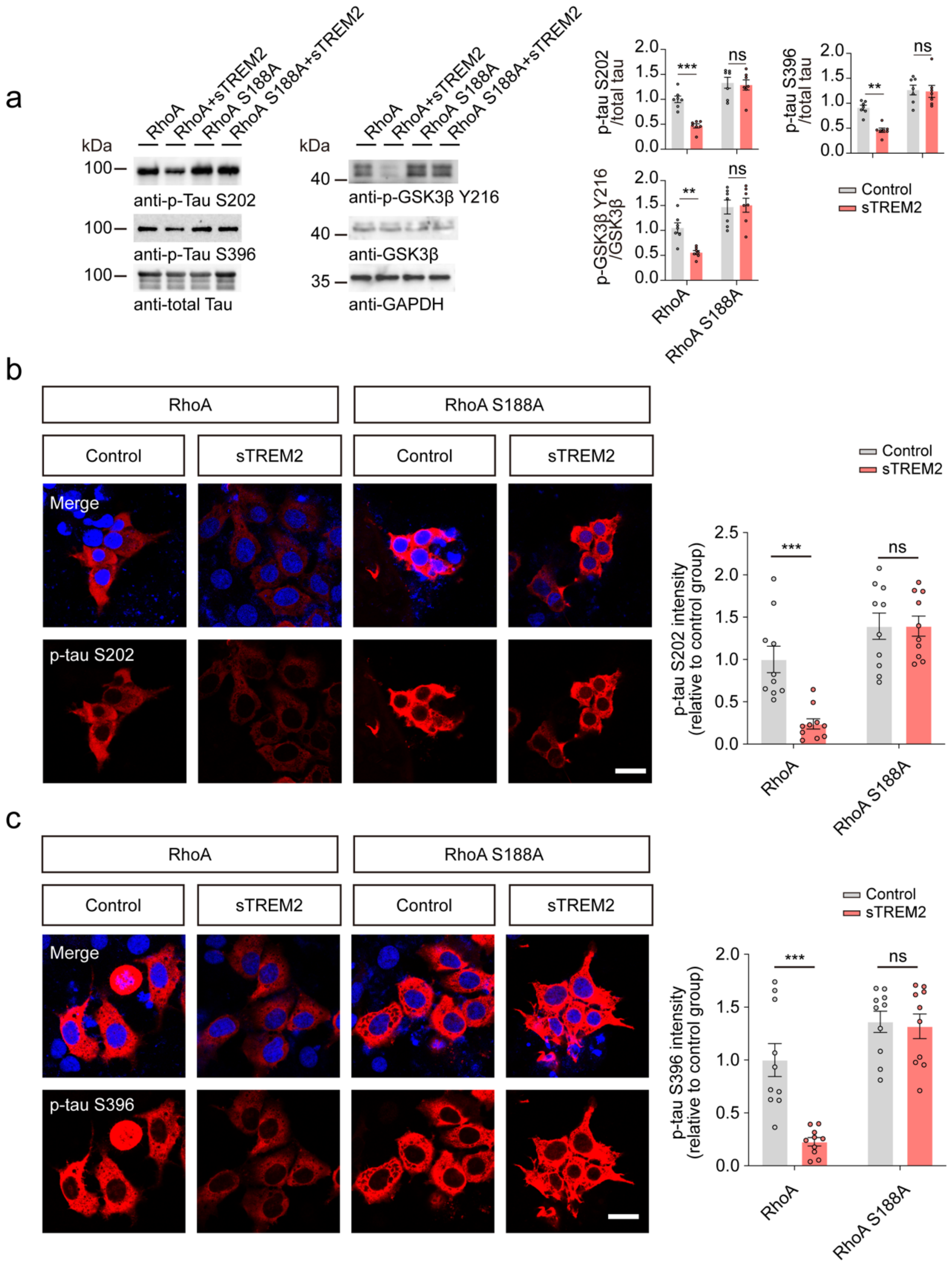

Supplementary Figure 7. RhoA S188A mutation blocks the effect of sTREM2 on tau phosphorylation. HEK293-Tau cells were transfected with wild-type or S188A mutant RhoA in an endogenous RhoA-null background, and the cells were treated with Fc (20 nM) or sTREM2 (20 nM) for 24 h. (**a**) Western blots and quantification showing the phosphorylation of tau and GSK3β (mean ± s.e.m.; n = 10 images of 5 independent experiments; ***P* < 0.01, ****P* < 0.001, two-way ANOVA). (**b, c**) p-Tau S202 (B) and p-tau S396 (c) immunostaining. (mean ± s.e.m.; two-way ANOVA, n = 10 images of 5 independent experiments; ****P* < 0.001). Scale bars, 25 μm.

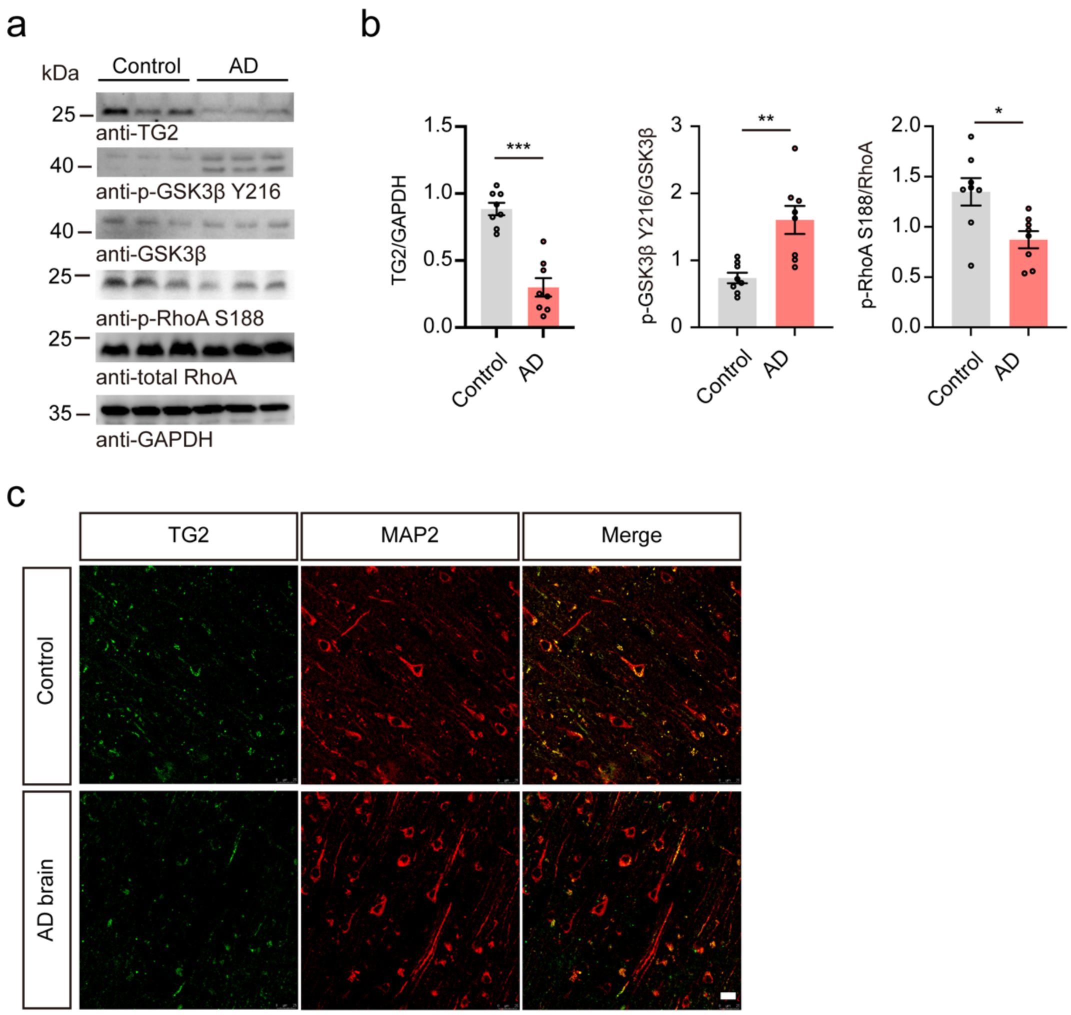

Supplementary Figure 8. The TG2/RhoA/GSK3β pathway in human AD patients. (**a**) Western blots showing the activation of the TG2/RhoA/GSK3β pathway in brain samples from AD and age-matched control subjects. (**b**) Quantification of the Western blot results (mean ± s.e.m.; n = 8 samples per group; *$P < 0.05$, **$P < 0.01$, ***$P < 0.001$. Student's *t*-test). (**c**) Immunostaining showing the expression of TG2 in the AD brain. Scale bar, 20 µm.

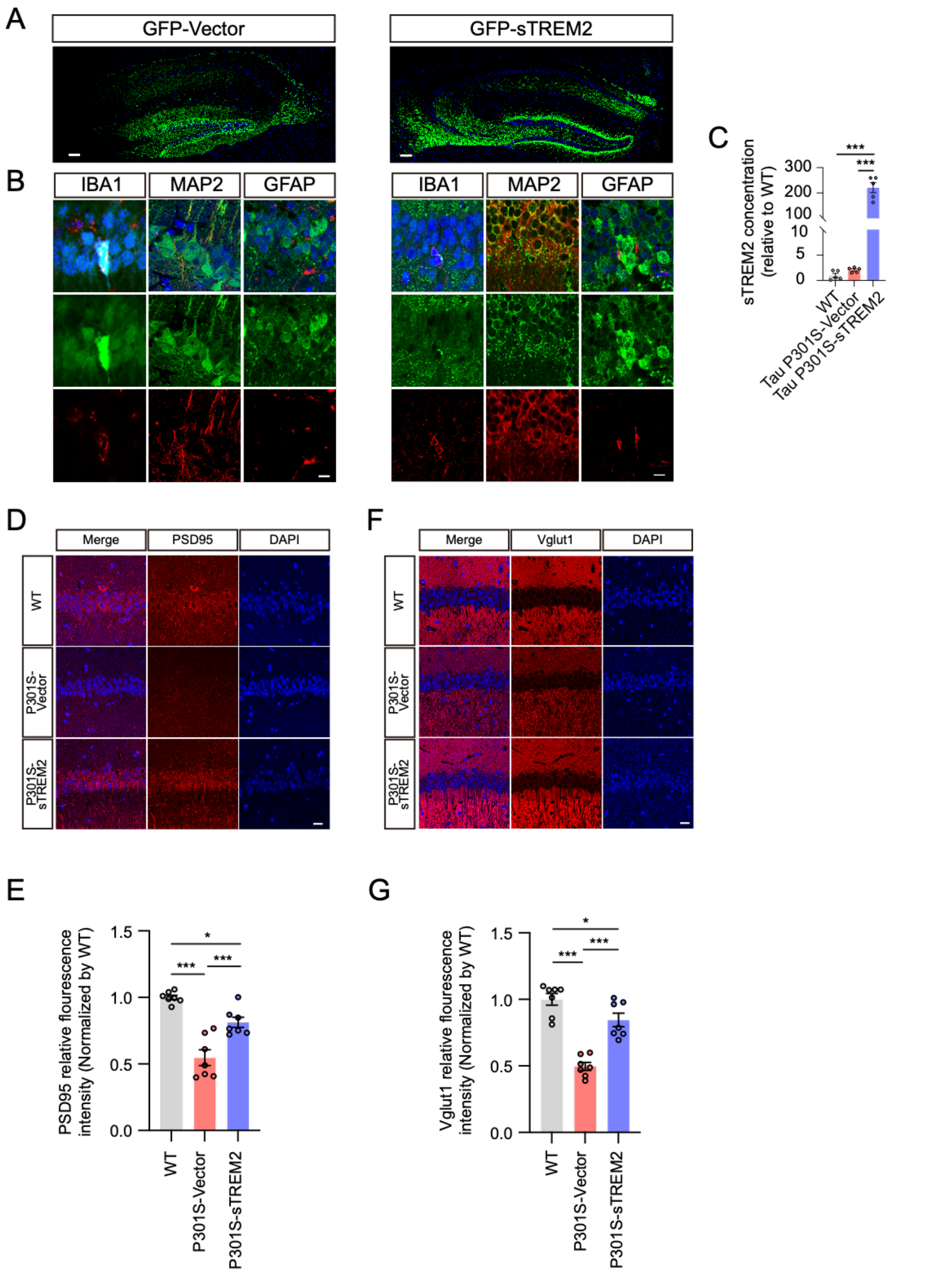

Supplementary Figure 9. sTREM2 protects synapses in Tau P301S mice. (**a**) Tau P301S mice were injected with AAV-EGFP-Vector or AAV-EGFP-sTREM2 at three months of age. Shown are the expression of EGFP and EGFP-sTREM2. Scale bars, 100 μm. n = 5 mice per group. (**b**) The expression of GFP and GFP-sTREM2 in microglia (stained with IBA1), neurons (stained with MAP2), and astrocytes (stained with GFAP). Scale bars, 10 μm. n = 5 mice per group. (**c**) The concentrations of sTREM2 detected

by ELISA in WT mice and tau P301S mice expressing EGFP-Vector or EGFP-sTREM2 (mean ± s.e.m.; n = 5 mice per group; compared with the control group, ***$P < 0.001$, one-way ANOVA). (**d-g**) Immunofluorescence and quantification of PSD95 (d, e) and Vglut1 (f, g) (mean ± s.e.m.; n = 7 mice per group; *$P < 0.05$, ***$P < 0.001$, one-way ANOVA). Scale bars, 20 μm.

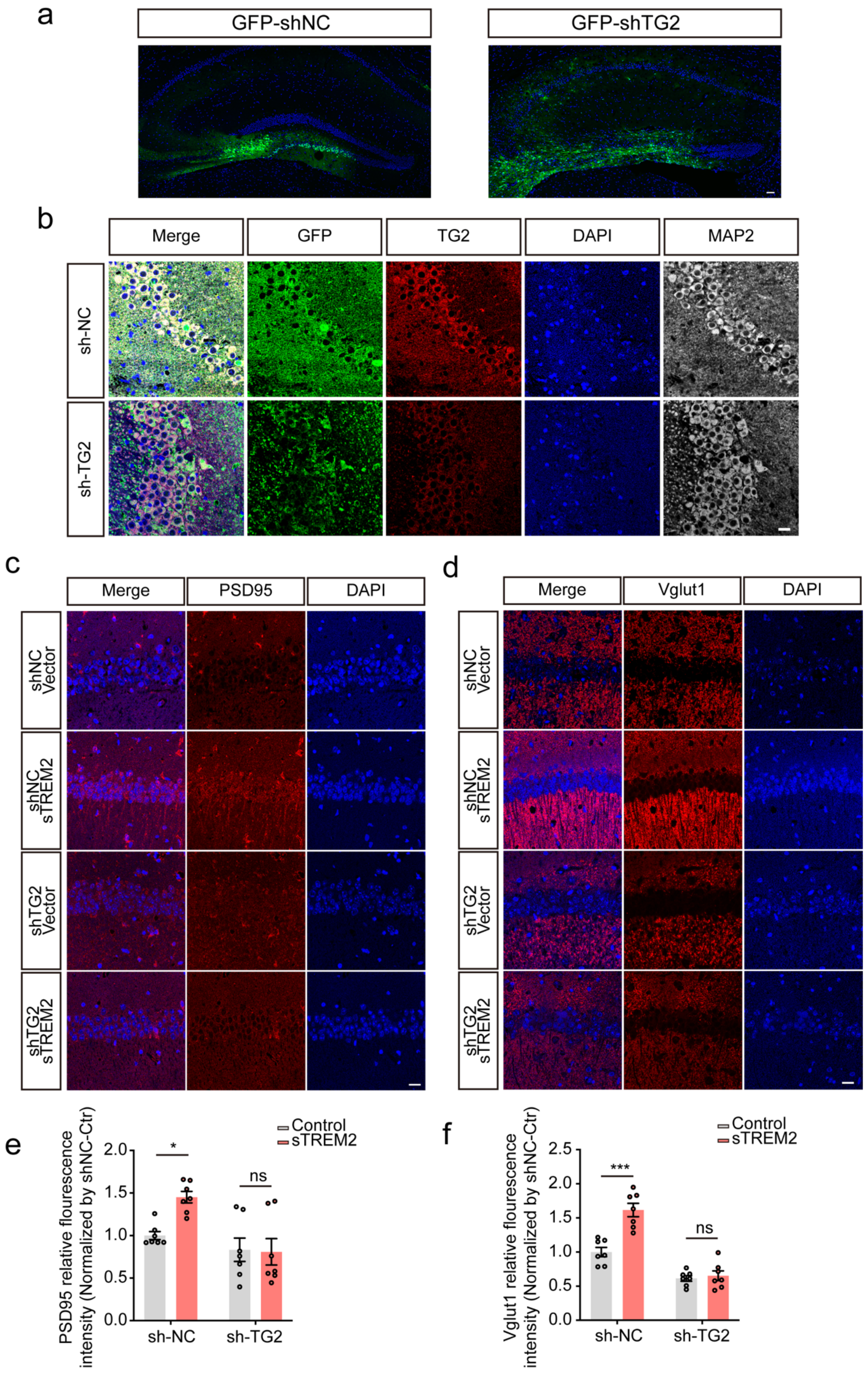

Supplementary Figure 10. The effect of TG2 knockdown on synapses *in vivo*. (a)

Tau P301S mice were injected with AAV-EGFP-shNC and AAV-EGFP-shTG2. Immunostaining of GFP showing the expression of AAV-EGFP-shNC and AAV-EGFP-shTG2. Scale bar, 100 μm. n = 7 mice per group. (**b**) Immunofluorescence showing the knockdown of TG2. n = 7 mice per group. (**c-f**) Immunofluorescence and quantification of PSD95 (d, e) and Vglut1 (f, g). Scale bars in B-D, 20 μm (mean ± s.e.m.; n = 7 mice per group; *$P < 0.05$, ***$P < 0.001$, one-way ANOVA).

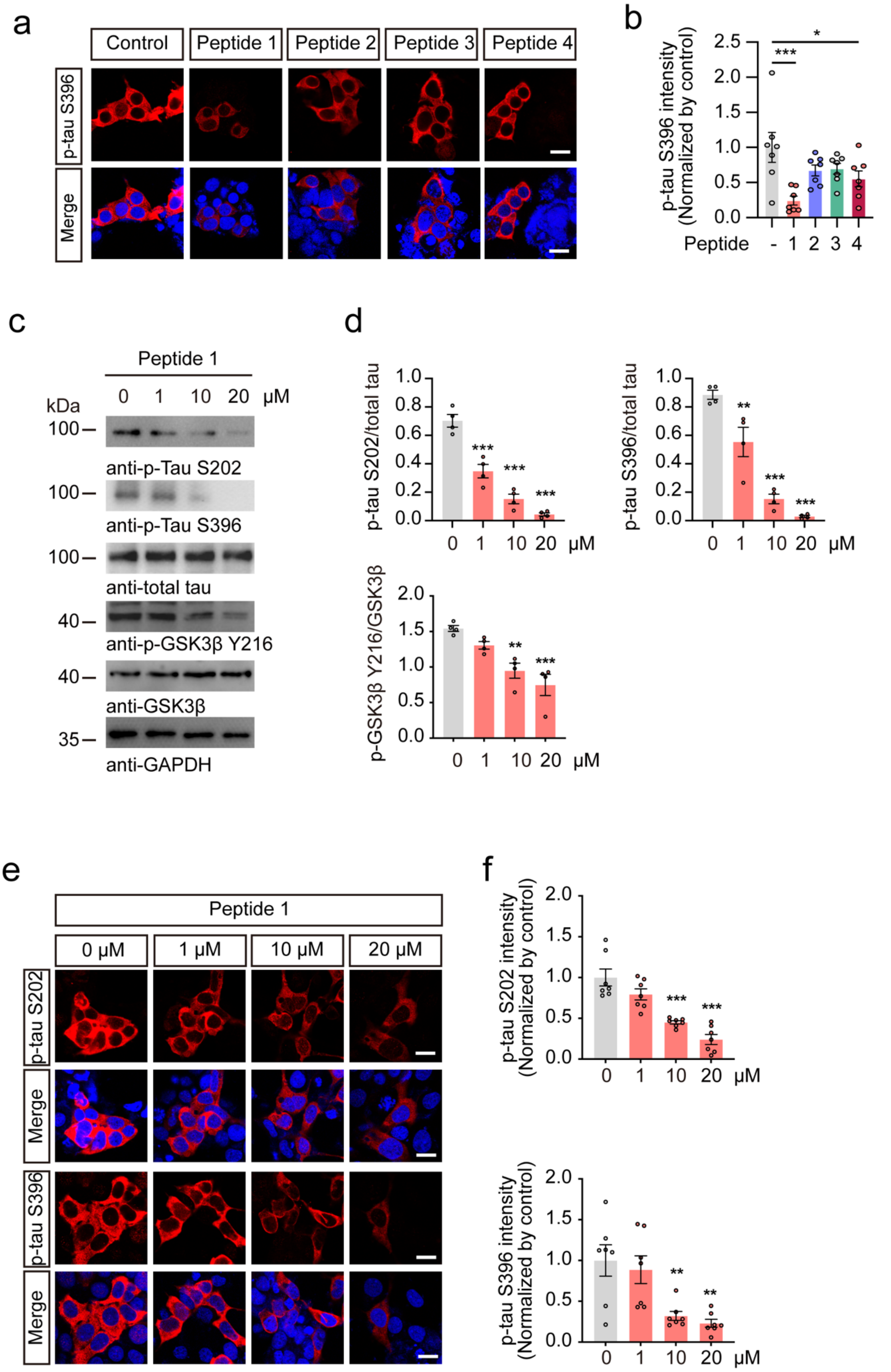

Supplementary Figure 11. Peptide 1 inhibits tau phosphorylation *in vitro*. (a, b) p-Tau S396 immunostaining (a) and quantification (b) of HEK293-tau cells treated with

different sTREM2 peptides for 24 h (mean ± s.e.m.; one-way ANOVA, n = 7 independent experiments; *$P < 0.05$, ***$P < 0.001$). Scale bars, 25 μm. (**c, d**) Western blots showing the phosphorylation of tau and GSK3β (mean ± s.e.m.; one-way ANOVA, n = 4 independent experiments; compared with the control group, **$P < 0.01$, ***$P < 0.001$). (**e, f**) p-Tau S202 and S396 immunostaining and quantification (mean ± s.e.m.; one-way ANOVA, n = 7 independent experiments; **$P < 0.01$, ***$P < 0.001$). Scale bars, 25 μm.

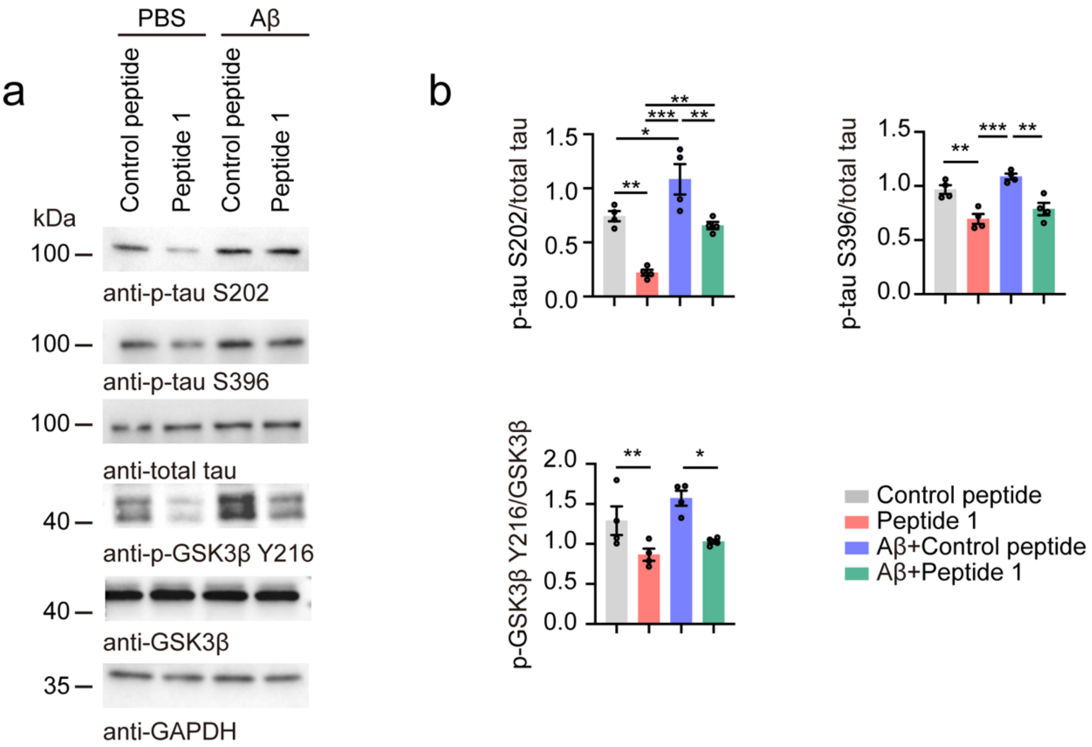

Supplementary Figure 12. Peptide 1 attenuates tau phosphorylation induced by Aβ oligomers. (a, b) Western blots and quantification showing the phosphorylation of tau and GSK3β in HEK293-tau cells exposed to Aβ (1 μM) in the presence or absence of peptide 1 (10 μM) for 24 h (mean ± s.e.m.; n = 4 per group; *$P < 0.05$, **$P < 0.01$, ***$P < 0.001$, one-way ANOVA).

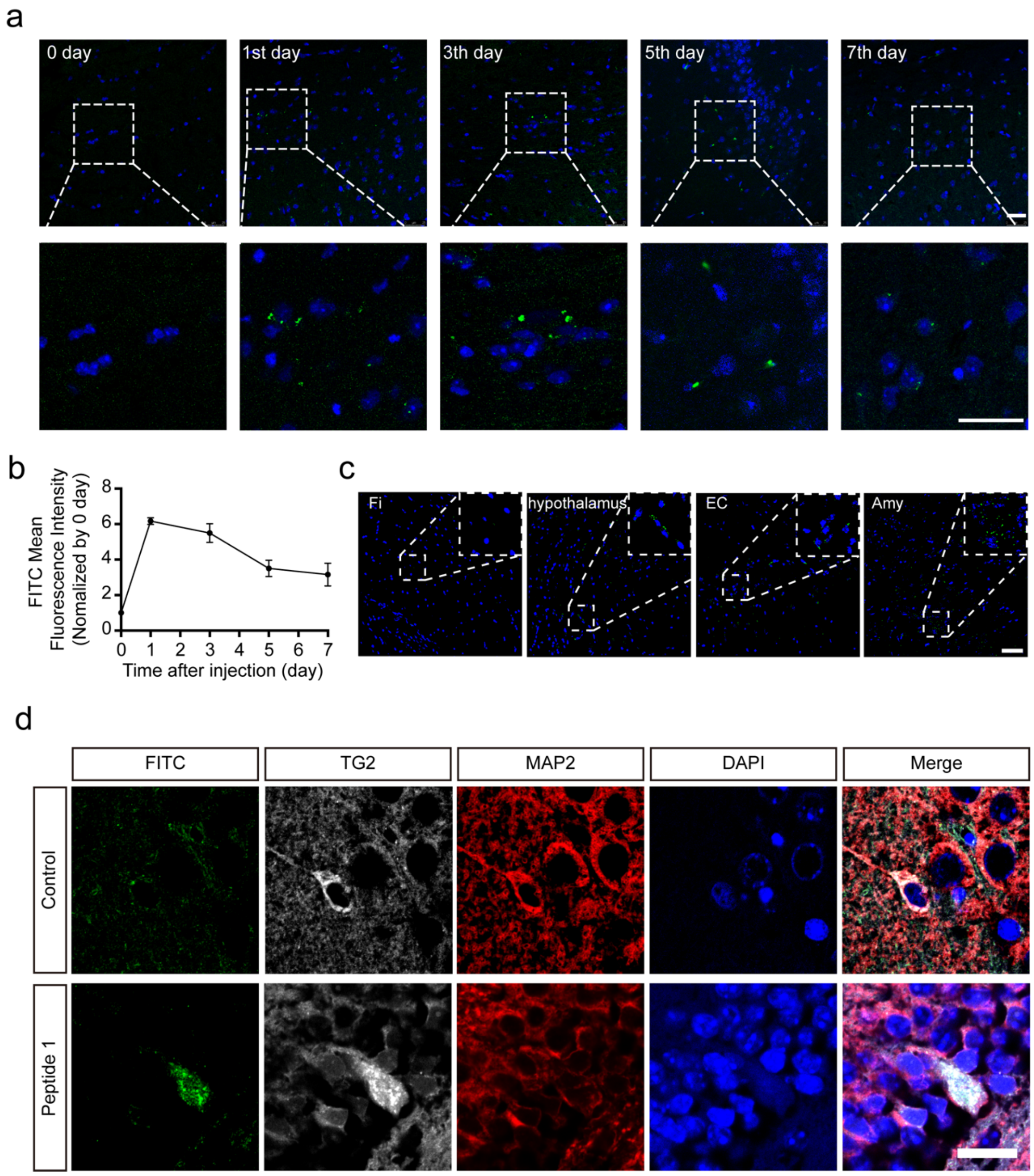

Supplementary Figure 13. The distribution of peptide 1 in the brains of mice administered with Tat-sTREM2 (77-89). (**a**) The mice were i.p. injected with FITC-labeled Tat-sTREM2 (77-89) (peptide 1) at 10 mg/kg. FITC fluorescence in the hippocampus was observed at 1, 3, 5, and 7 days after injection. Scale bars, 25 µm. n = 4 mice per group. (**b**) Quantification of fluorescence (mean ± s.e.m.; n = 4 mice per group). (**c**) The distribution of FITC-peptide 1 in different brain areas. Scale bars, 50 µm. n = 4 mice per group. (**d**) FITC-peptide 1 colocalized with TG2 in neurons. Immunostaining of FITC, TG2, and MAP2 in tau P301S mice injected with FITC-control peptide or FITC-peptide1. n = 4 mice per group. Scale bars, 20 µm. Fi, fimbria; EC, entorhinal cortex, Amy, amygdala.

Supplementary table 1. The membrane proteins that bound to sTREM2 as identified by LC-MS/MS.

Accession	Gene	spectra number (Fc-sTREM2)	spectra number (Fc-Vector)	Log2(Fc-sTREM2/Fc)	Log2(MeanSP)	Diff Sig
**P10909**	CLU	22	1	4.4594	3.5236	++
**Q13885**	TUBB2A	13	1	3.7004	2.8074	++
**Q9BVA1**	TUBB2B	13	1	3.7004	2.8074	++
**P02765**	AHSG	12	2	2.585	2.8074	++
**Q08380**	LGALS3BP	6	1	2.585	1.8074	++
**P37802**	TAGLN2	5	1	2.3219	1.585	++
**P01023**	A2M	4	1	2	1.3219	+
**P07237**	P4HB	4	1	2	1.3219	+
**P22314**	UBA1	4	1	2	1.3219	+
**Q9Y4L1**	HYOU1	4	1	2	1.3219	+
**P06733**	ENO1	7	3	1.2224	2.3219	+
**P11021**	HSPA5	23	10	1.2016	4.0444	+
**P10809**	HSPD1	5	2	1.3219	1.8074	+
**P13639**	EEF2	15	6	1.3219	3.3923	+
**P02768**	ALB	7	3	1.2224	2.3219	+
**Q14974**	KPNB1	8	4	1	2.585	+

Fc-tagged sTREM2 was incubated with membrane fractions of SH-SY5Y cells. The protein was purified using protein A/G beads. LC-MS/MS identified the bait protein (sTREM2) and the proteins that interact with sTREM2. The mass spectrometry data generated by TripleTOF5600 were retrieved through ProteinPilot (V4.5) using the database retrieval algorithm Paragon. The proteins were screened based on the spectrum number for each protein. The protein with a spectrum number of 0 was artificially filled with 1 to reduce false-positive results caused by the identification of low-abundance proteins. The spectra number ratio of the Fc-sTREM2 group to the Fc-Vector group, and the average number of spectra (MeanSP) were calculated. The differential protein screening boundary line $y = c/(x-x_0)$ was set, where $x = \log_2$ratio, $y = \log_2$MeanSP, c and x_0 were the boundary line parameters. The former $c = 1$, $x_0 = \log_2 1.5$, the latter $c = \log_2 2.5$, $x_0 = 1$.

Supplementary table 2. Tagln2 (TG2) on the membrane of wild-type neurons was detected to interact with sTREM2.

Accession	Gene	Intensity (control)	Intensity (sTREM2)	sTREM2/ control	Log2 (sTREM2/control)
Q9WVA4	Tagln2	9.76	15.62	58.32	5.87

Membrane proteins from wild-type primary neurons were incubated with sTREM2. sTREM2-binding proteins were analyzed by LC-MS/MS. The data were retrieved using

MaxQuant (V1.6.6) software with Andromeda as the database retrieval algorithm. The signal intensity detected by mass spectrometry of the TG2 peptide segment was shown.

Supplementary table 3. Tagln2 (TG2) on the membrane of tau P301S neurons was detected to interact with sTREM2.

Accession	Gene	Intensity (control)	Intensity (sTREM2)	sTREM2/control	Log2 (sTREM2/control)
**Q9WVA4**	Tagln2	8.71	15.47	108.03	6.76

Membrane proteins from tau P301S primary neurons were incubated with sTREM2. sTREM2-binding proteins were analyzed by LC-MS/MS. Mass spectrometry data were retrieved using MaxQuant (V1.6.6) software with Andromeda as the database retrieval algorithm. The signal intensity detected by mass spectrometry of the TG2 peptide segment was shown.
