# Supplementary material for: Soluble TREM2 ameliorates tau phosphorylation and cognitive deficits through activating transgelin-2 in Alzheimer’s disease
Source: Nat Commun. 2023 Oct 21;14:6670. doi: 10.1038/s41467-023-42505-x (PMC10590452; doi:10.1038/s41467-023-42505-x)
Supplement: Supplementary file 3 — Reporting Summary [file 41467_2023_42505_MOESM3_ESM.pdf]

nature portfolio

Corresponding author(s): Zhentao Zhang
Last updated by author(s): Sep 24, 2023

Reporting Summary

Nature Portfolio wishes to improve the reproducibility of the work that we publish. This form provides structure for consistency and transparency in reporting. For further information on Nature Portfolio policies, see our Editorial Policies and the Editorial Policy Checklist.

Statistics

For all statistical analyses, confirm that the following items are present in the figure legend, table legend, main text, or Methods section.

n/a	Confirmed	
☐	☒	The exact sample size (*n*) for each experimental group/condition, given as a discrete number and unit of measurement
☐	☒	A statement on whether measurements were taken from distinct samples or whether the same sample was measured repeatedly
☐	☒	The statistical test(s) used AND whether they are one- or two-sided<br>*Only common tests should be described solely by name; describe more complex techniques in the Methods section.*
☐	☒	A description of all covariates tested
☐	☒	A description of any assumptions or corrections, such as tests of normality and adjustment for multiple comparisons
☐	☒	A full description of the statistical parameters including central tendency (e.g. means) or other basic estimates (e.g. regression coefficient) AND variation (e.g. standard deviation) or associated estimates of uncertainty (e.g. confidence intervals)
☐	☒	For null hypothesis testing, the test statistic (e.g. *F*, *t*, *r*) with confidence intervals, effect sizes, degrees of freedom and *P* value noted<br>*Give P values as exact values whenever suitable.*
☒	☐	For Bayesian analysis, information on the choice of priors and Markov chain Monte Carlo settings
☒	☐	For hierarchical and complex designs, identification of the appropriate level for tests and full reporting of outcomes
☐	☒	Estimates of effect sizes (e.g. Cohen's *d*, Pearson's *r*), indicating how they were calculated

Our web collection on statistics for biologists contains articles on many of the points above.

Software and code

Policy information about availability of computer code

Data collection	Transmission electron microscopy (hitachi), confocal laser microscope (LEICA), Spectra Max plate reader (Molecular Devices, Sunnyvale CA), Olympus DP80 microscope equipped with TH4-200 and U-HGLGPS light sources
Data analysis	GraphPad Prism (version 8.0);ImageJ software(version 2.1.0/1.53c) Imagej software (version 2.1.0/1.53c) ANY-Maze software (San Diego Instruments)

For manuscripts utilizing custom algorithms or software that are central to the research but not yet described in published literature, software must be made available to editors and reviewers. We strongly encourage code deposition in a community repository (e.g. GitHub). See the Nature Portfolio guidelines for submitting code & software for further information.

Data

Policy information about availability of data

All manuscripts must include a data availability statement. This statement should provide the following information, where applicable:

- Accession codes, unique identifiers, or web links for publicly available datasets
- A description of any restrictions on data availability
- For clinical datasets or third party data, please ensure that the statement adheres to our policy

All data in this study are included in the manuscript and supporting files. The Mus musculus Proteome Reference Database was from UP000000589 [https://

www.uniprot.org/proteomes/UP000000589], while the Homo sapiens Proteome Reference Database was from UP000005640 [https://www.uniprot.org/proteomes/UP000005640]. The raw data generated in this study are provided in the Source Data file.

Research involving human participants, their data, or biological material

Policy information about studies with human participants or human data. See also policy information about sex, gender (identity/presentation), and sexual orientation and race, ethnicity and racism.

Reporting on sex and gender	Donors were 44% female and 56% male. Gender information was not reported.
Reporting on race, ethnicity, or other socially relevant groupings	The human brain samples of AD patients and age-matched controls for western blotting were obtained from the Xiangya School of Medicine, with individuals primarily of Asian ancestry. The brain tissues for immunostaining were obtained from the Emory Alzheimer's Disease Research Center (ADRC) Brain Bank, with individuals primarily of Caucasian ancestry.
Population characteristics	The diagnosis of AD was confirmed by the presence of amyloid plaques and neurofibrillary tangles. The age at death of patients ranged between 52 and 70 years old. The post-mortem intervals (PMI) were 2.5 to 9 h.
Recruitment	Participants were research volunteers in Xiangya School of Medicine or ,the Emory Alzheimer's Disease Research Center (ADRC) who consented to donate their brains for research.
Ethics oversight	Post-mortem brain samples were collected with approved consent of Xiangya School of Medicine and the Emory Alzheimer's Disease Research Center (ADRC).

Note that full information on the approval of the study protocol must also be provided in the manuscript.

Field-specific reporting

Please select the one below that is the best fit for your research. If you are not sure, read the appropriate sections before making your selection.

☒ Life sciences ☐ Behavioural & social sciences ☐ Ecological, evolutionary & environmental sciences

For a reference copy of the document with all sections, see nature.com/documents/nr-reporting-summary-flat.pdf

Life sciences study design

All studies must disclose on these points even when the disclosure is negative.

Sample size	The sample size was determined by Power and Precision software (Biostat).
Data exclusions	No data points were excluded from statistical analysis.
Replication	We replicated the experiments three times at least, achieving similar results and standard deviations were within expected ranges.
Randomization	The mice were randomized into different groups by using a random number table. For other experiments, samples were also randomly allocated into experimental groups.
Blinding	Investigators were blinded to the group allocation during the data collection and analyses.

Reporting for specific materials, systems and methods

We require information from authors about some types of materials, experimental systems and methods used in many studies. Here, indicate whether each material, system or method listed is relevant to your study. If you are not sure if a list item applies to your research, read the appropriate section before selecting a response.

Materials & experimental systems

n/a	Involved in the study	
☐	☒	Antibodies
☐	☒	Eukaryotic cell lines
☒	☐	Palaeontology and archaeology
☐	☒	Animals and other organisms
☒	☐	Clinical data
☒	☐	Dual use research of concern
☒	☐	Plants

Methods

n/a	Involved in the study	
☒	☐	ChIP-seq
☒	☐	Flow cytometry
☒	☐	MRI-based neuroimaging

Antibodies

Antibodies used

Anti-p-tau S202 (ab108387), p-tau S396 (ab109390), p-GSK3β Y216 (ab75745), GSK3β (ab32391), and p-RhoA S188 (ab41435) were purchased from Abcam (Cambridge, UK). Anti-GST (10000-0-AP), GFP (66002-2-Ig), His (66005-1-Ig), RhoA (10749-1-AP), GAPDH (60004-1-Ig), and TG-2 (60044-1-Ig), TG-2 (10234-2-AP), anti-human IgG (16402-1-AP) were purchased from Proteintech. The anti-TG-2 (sc-166697) antibody was from Santa Cruz. Tau5 antibody (AHB0042), anti-p-Tau (Ser202, Thr205) (MN1020), anti-p-tau T181 (MN1050), Goat anti-Mouse IgG (H+L) Highly Cross-Adsorbed Secondary Antibody, Alexa Fluor 488 (A-11001), Goat anti-Rabbit IgG (H+L) Highly Cross-Adsorbed Secondary Antibody, Alexa Fluor 488 (A-11034), Goat anti-Rabbit IgG (H+L) Cross-Adsorbed Secondary Antibody, Alexa Fluor 594 (A-11012), Goat anti-Mouse IgG (H+L) Cross-Adsorbed Secondary Antibody, Alexa Fluor 594 (A-11005) were purchased from ThermoFisher. Anti p-tau S404 (310196), and p-tau T231 (381181) were from Zenbio.

Validation

Anti-p-tau S202 (ab108387), Abcam
Vendor information: https://www.abcam.com/tau-phospho-s202-antibody-epr2402-ab108387.html
Application: western blot (1:1000, validated by the correspondent manufacturers), immunofluorescence (1:1000, validated by PMID: 32982685)

Anti-p-tau S396 (ab109390), Abcam
Vendor information: https://www.abcam.com/tau-phospho-s396-antibody-epr2731-ab109390.html
Application: western blot (1:1000, validated by the correspondent manufacturers), immunofluorescence (1:1000, validated by PMID: 31263400, 28947735)

Anti-p-GSK3β Y216 (ab75745), Abcam
Vendor information: https://www.abcam.com/gsk3-beta-phospho-y216--gsk3-alpha-phospho-y279-antibody-ab75745.html
Application: western blot (1:1000, validated by the correspondent manufacturers)

Anti-GSK3β (ab32391), Abcam
Vendor information: https://www.abcam.com/gsk3-beta-antibody-y174-ab32391.html
Application: western blot (1:1000, validated by the correspondent manufacturers)

Anti-p-RhoA S188 (ab41435), Abcam
Vendor information: https://www.abcam.com/rhoa-phospho-s188-antibody-ab41435.html
Application: western blot (1:500, validated by the correspondent manufacturers)

Anti-GST (10000-0-AP), Proteintech
Vendor information: https://www.ptgcn.com/products/gst-Antibody-10000-0-AP.htm
Application: western blot (1:5000, validated by the correspondent manufacturers)

Anti-GFP (66002-2-Ig), Proteintech
Vendor information: https://www.ptgcn.com/products/eGFP-Antibody-66002-1-Ig.htm
Application: western blot (1:5000, validated by the correspondent manufacturers)

Anti-His (66005-1-Ig), Proteintech
Vendor information: https://www.ptgcn.com/products/His-Tag-Antibody-66005-1-Ig.htm
Application: western blot (1:5000, validated by the correspondent manufacturers)

Anti-RhoA (10749-1-AP), Proteintech
Vendor information: https://www.ptgcn.com/products/RHOA-Antibody-10749-1-AP.htm
Application: western blot (1:500, validated by the correspondent manufacturers)

Anti-GAPDH (60004-1-Ig), Proteintech
Vendor information: https://www.ptgcn.com/products/GAPDH-Antibody-60004-1-Ig.htm
Application: western blot (1:10000, validated by the correspondent manufacturers)

Anti-TG-2(60044-1-Ig), Proteintech
Vendor information: https://www.ptglab.com/products/TAGLN2-Antibody-60044-1-Ig.htm
Application: western blot, immunofluorescence (1:500, validated by the correspondent manufacturers)

Anti-TG-2 (10234-2-AP), Proteintech
Vendor information: https://www.ptgcn.com/products/TAGLN2-Antibody-10234-2-AP.htm
Application: western blot, immunofluorescence (1:500, validated by the correspondent manufacturers)

anti-human IgG (16402-1-AP), Proteintech
Vendor information: https://www.ptgcn.com/products/IGHG4-Antibody-16402-1-AP.htm#publications
Application: immunofluorescence (1:200, validated by the correspondent manufacturers);western blot (1:1000, validated by the correspondent manufacturers)

anti-TG-2 (sc-166697) , Santa Cruz
Vendor information: https://www.scbt.com/p/transgelin-2-antibody-g-5
Application: immunofluorescence (1:200, validated by the correspondent manufacturers)

Tau5 antibody (AHB0042), ThermoFisher
Vendor information: https://www.thermofisher.cn/cn/zh/antibody/product/Tau-Antibody-clone-TAU-5-Monoclonal/AHB0042
Application: western blot (1:1000, validated by the correspondent manufacturers)

anti-p-Tau (Ser202, Thr205) (MN1020)
Vendor information: https://www.thermofisher.cn/cn/zh/antibody/product/Phospho-Tau-Ser202-Thr205-Antibody-clone-AT8-Monoclonal/MN1020
Application: immunohistochemistry (1:1000, validated by the correspondent manufacturers)

anti-p-tau T181 (MN1050)
Vendor information: https://www.thermofisher.cn/cn/zh/antibody/product/Phospho-Tau-Thr181-Antibody-clone-AT270-Monoclonal/MN1050
Application: western blot (1:1000, validated by the correspondent manufacturers)
Goat anti-Mouse IgG (H+L) Highly Cross-Adsorbed Secondary Antibody, Alexa Fluor 488 (A-11001), ThermoFisher
Vendor information: https://www.thermofisher.cn/cn/zh/antibody/product/Goat-anti-Mouse-IgG-H-L-Cross-Adsorbed-Secondary-Antibody-Polyclonal/A-11001
Application: immunofluorescence (1:1000, validated by the correspondent manufacturers)

Goat anti-Rabbit IgG (H+L) Highly Cross-Adsorbed Secondary Antibody, Alexa Fluor 488 (A-11034), ThermoFisher
Vendor information: https://www.thermofisher.cn/cn/zh/antibody/product/Goat-anti-Rabbit-IgG-H-L-Highly-Cross-Adsorbed-Secondary-Antibody-Polyclonal/A-11034
Application: immunofluorescence (1:1000, validated by the correspondent manufacturers)

Goat anti-Rabbit IgG (H+L) Cross-Adsorbed Secondary Antibody, Alexa Fluor 594 (A-11012), ThermoFisher
Vendor information: https://www.thermofisher.cn/cn/zh/antibody/product/Goat-anti-Rabbit-IgG-H-L-Cross-Adsorbed-Secondary-Antibody-Polyclonal/A-11012
Application: immunofluorescence (1:1000, validated by the correspondent manufacturers)

Goat anti-Mouse IgG (H+L) Cross-Adsorbed Secondary Antibody, Alexa Fluor 594 (A-11005), ThermoFisher
Vendor information: https://www.thermofisher.cn/cn/zh/antibody/product/Goat-anti-Mouse-IgG-H-L-Cross-Adsorbed-Secondary-Antibody-Polyclonal/A-11005
Application: immunofluorescence (1:1000, validated by the correspondent manufacturers)

anti p-tau S404 (310196)
Vendor information: http://www.zen-bio.cn/prod_view.aspx?IsActiveTarget=True&TypeId=180&Id=540405&FId=t3:180:3
Application: western blot (1:1000, validate by the correspondent manufacturers)

anti-p-tau T231 (381181)
Vendor information: http://www.zen-bio.cn/prod_view.aspx?IsActiveTarget=True&TypeId=171&Id=565842&FId=t3:171:3
Application: western blot (1:1000, validate by the correspondent manufacturers)

Eukaryotic cell lines

Policy information about cell lines and Sex and Gender in Research

Cell line source(s): All cell lines including SH-SY5Y, HEK293 and BV2 were obtained from the American Tissue Culture Collection (ATCC). HEK293 cells were used for transient transfections using polyethyleneimine (PEI). The HEK293 cell line stably overexpressing GFP-Tau (HEK293-Tau cells) was established via LV-EF1a-EGFP-tau (1-441 aa) infection and puromycin selection.

Authentication: We did not perform additional authentication of this cell line

Mycoplasma contamination: The cell line was tested negative for mycoplasma.

Commonly misidentified lines (See ICLAC register): No commonly misidentified cell lines were used

Animals and other research organisms

Policy information about studies involving animals; ARRIVE guidelines recommended for reporting animal research, and Sex and Gender in Research

Laboratory animals: Tau P301S mice (stock number: 008169) and wild-type C57BL/6J mice (stock number: 000664) were from the Jackson Laboratory. All mice were kept under specific pathogen-free (SPF) conditions in a 14 h light/10 h dark cycle and had free access to food and water. Three-month-old tau P301S mice or wild-type C57BL/6J mice were anesthetized and stereotactically injected with virus or i.p. administered peptides. The mice were analyzed at 7 months of age.

Wild animals: The study did not involve wild animals.

Reporting on sex: Only male mice were used in this study.

Field-collected samples: The study did not involve samples collected from the field.

Ethics oversight: The protocol was reviewed and approved by the Animal Care and Use Committee of Renmin Hospital of Wuhan University.

Note that full information on the approval of the study protocol must also be provided in the manuscript.
